# Mid-Term Results of Single-Stage Surgery for Patients with Chronic Osteomyelitis Using Antibiotic-Loaded Resorbable PerOssal^®^ Beads

**DOI:** 10.3390/microorganisms11071623

**Published:** 2023-06-21

**Authors:** Andrea Sambri, Luca Cevolani, Valentina Passarino, Marta Bortoli, Stefania Claudia Parisi, Michele Fiore, Laura Campanacci, Eric Staals, Davide Maria Donati, Massimiliano De Paolis

**Affiliations:** 1 Orthopedic and Traumatology Unit, IRCCS Azienda Ospedaliero-Universitaria di Bologna, 40138 Bologna, Italy; valentina.passarino@studio.unibo.it (V.P.); marta.bortoli@ior.it (M.B.); stefaniaclaudia.parisi@hotmail.it (S.C.P.); michele.fiore@ior.it (M.F.); massimiliano.depaolis@aosp.bo.it (M.D.P.); 2 Orthopedic Oncology Unit, IRCCS Istituto Ortopedico Rizzoli, 40136 Bologna, Italy; luca.cevolani@ior.it (L.C.); laura.campanacci@ior.it (L.C.); eric.staals@ior.it (E.S.); davide.donati@ior.it (D.M.D.)

**Keywords:** perossal, chronic osteomyelitis, bone filler, antibiotic, calcium sulfate

## Abstract

This retrospective study reports on the treatment of chronic osteomyelitis with local debridement combined with PerOssal^®^. The diagnosis of chronic osteomyelitis was confirmed in all cases and classified according to the Cierny–Mader (C-M) classification. The primary outcome was the eradication of infection at a minimum of one year after surgery. A total of 93 patients (median age: 40 years) were included. The most represented sites were the femur (24, 25.8%) and tibia (52, 55.9%). Twenty-six patients (28.0%) had significant local or systemic comorbidities (C-M Class B hosts). According to anatomic type, 31 cases were type I, 13 type II, 21 type III and 28 type IV. Vancomycin was added to PerOssal^®^ in most cases (80, 86.0%). In 24 (25.8%) cases, Vancomycin and Rifampicin were combined. In 32 (34.4%) cases, intraoperative cultures were negative. *Staphylococcus aureus* was isolated in 39 (63.9%) patients, and Gram-negative bacteria were isolated in 12 cases. The median follow-up was 21 months (range 12–84). A total of 21 (22.6%) patients developed an infection recurrence (IR) after a median follow-up of 11 months (range: 1–47). PerOssal^®^ holds several practical advantages compared to other bone void fillers. Thus, due to its good biocompatibility and sufficient antibiotic release, it represents a viable adjuvant treatment in chronic osteomyelitis.

## 1. Introduction

Chronic osteomyelitis (COM) is a devastating complication following trauma or orthopedic surgery [1], which is characterized by low-grade inflammation, the presence of sequestrum (necrotic and dead bone) and fistulous tracts [2]. The devascularized nature of the sequestrum may protect bacteria from host immune response, thus limiting the effectiveness of many antibiotics [3].

The treatment of COM is complex and requires a dedicated multidisciplinary team [4,5]. It often requires multiple surgical interventions and a prolonged course of antibiotic therapy [6,7]. The debridement of non-vital, infected and fibrous tissue along with osseous structures is required, often resulting in large-bone and soft-tissue defects [8,9,10,11]. After debridement, the cavitary bone defect can be filled with antibiotic-loaded polymethyl methacrylate (PMMA) beads. However, problems related to PMMA include possible biofilm formation on the bone cement or a second procedure to remove PMMA. These problems can be avoided by using synthetic, antibiotic-loaded, resorbable bone void fillers (BVF) [12]. Implanting antibiotic carriers directly at the site of the infection, where they locally release an active agent, has been proven to be a reliable option in the treatment of COM. They allow for a reduced number of surgical procedures, a shortened hospitalization period and fewer systemic side effects when compared to parenteral antibiotic therapy alone [13,14]. However, bone formation in defects caused after the excision of infected bone with calcium sulphate (CS) BVF is unreliable, with pathological fractures occurring in 5% to 8% of patients [15,16].

PerOssal^®^ (OSARTIS, Münster, Hesse, Germany) was developed, consisting of a combination of CS and nanocrystalline hydroxyapatite (HA). By adding HA, biocompatibility increased, which was demonstrated in vitro and in vivo [17]. PerOssal^®^ can be loaded with several commercially available antibiotics depending on the specific antimicrobial sensitivity of the isolated bacteria.

The aim of our study is to assess the outcomes of local debridement combined with PerOssal^®^ as a single-stage treatment of localized COM in order to analyze the complications and recurrence of infection.

## 2. Methods

We present a retrospective multi-center cohort study performed between January 2012 and January 2021. We recruited patients with a confirmed COM treated at two different centers, with a comparable diagnostic process, treatment algorithm and follow-up schedule.

The inclusion criteria for enrolling patients were as follows: patients with proven COM who underwent surgery with fenestration and debridement, combined with the placement of PerOssal^®^ beads; patients for whom clinical, radiological and microbiological records were complete; patients with at least 12 months of follow-up; and patients who provided informed consent. Exclusion criteria included infected non-unions, diabetic foot infections, patients with calcium metabolism disorders or a known allergy to CS or HA, patients with renal failure and unfit patients (Cierny–Mader class C hosts).

The diagnosis of COM was carried out according to the method of Glaudemans et al. [18]. Chronic osteomyelitis was defined as having symptoms for a minimum of six months with clinical and radiological features accompanied by at least one of the following: the presence of a sinus, an abscess or intra-operative pus, supportive histology or two or more positive microbiological cultures [19]. When cultures were negative, a patient was only included in the study when there was positive histology, with the presence of a draining sinus or intra-operative pus.

All patients were preoperatively evaluated using X-rays, magnetic resonance imaging (MRI) or computerized tomography (CT) of the involved bone [20]. Osteomyelitis was classified according to Cierny–Mader (C-M) classification [21]: where type I represents medullary, type II superficial, type III localized and type IV diffuse osteomyelitis. An A-host means a normal host, and a B-host can be locally compromised (Bl), systemically compromised (Bs) or both (Bls). The C-host means that the treatment can be worse than the disease itself.

Debridement aimed to remove all infected or necrotic bone and soft tissue, often fully identified at the time of surgery [8]. A thorough debridement was carried out, and all devitalized bone was removed with curettes and a high-speed burr. The remaining cavity, after the excision of infected bone, was washed then irrigated with a hydrogen peroxide solution and dried using gauze packing. The dry cavity was filled with PerOssal^®^ beads, composed of 51.5% of nanocrystalline HA and 48.5% of CS [22]. PerOssal^®^ was loaded with antibiotic drugs based on previous culture samples or epidemiology data [22,23].

Empirical intravenous broad-spectrum antibiotic therapy started immediately following surgery, after at least five tissue samples were taken from representative areas and sent to be cultured. All patients were managed in collaboration with a dedicated infectious diseases specialist. After culture results, antibiotic therapy was de-escalated, using an oral regimen whenever possible. Antibiotics were continued for 4–6 weeks, until the normalization of the C-reactive protein (CRP).

Bone defect filling was assessed using X-rays at 6 and 12 months of follow-up in all cases with radiographs available for retrospective analysis. [16] The primary outcome was the eradication of infection at a minimum of one year after surgery. Failure of treatment was defined as recurrent infection with further surgery performed for infection, recurrent sinus formation or patients requiring antibiotic treatment for persisting symptoms [16]. The Kaplan–Meier method was used as a univariate, unadjusted analysis of prognostic factors to estimate the infection recurrence (IR) rate. The estimated IR interval was defined as the time between surgery and IR. Being a retrospective study, patients were censored at the last available follow-up. Differences in survival rates were assessed using the log-rank test, and *p* values < 0.05 were considered significant. All analyses were completed using the Statistical Package for Social Science (IBM Corp, Armonk, NY, USA. Released 2013. IBM SPSS Statistics for Windows, Version 22.0. IBM Corp: Armonk, NY, USA). For any wound healing problems, pathological fractures and need for re-operation were considered as secondary outcomes.

## 3. Results

A total of 123 consecutive patients were found in the prospectively maintained databases of our institutions. However, 30 patients were excluded because they did not fit with the inclusion criteria. Thus, a total of 93 consecutive patients (68 male, 25 female) with confirmed COM were included. (Table 1) The median age was 40 years (4 to 73).

Osteomyelitis followed an open fracture or the internal fixation of closed fractures in 25 patients (26.9%). There were 47 patients (50.5%) who had hematogenous osteomyelitis and 21 (22.6%) with infections after orthopedic surgery.

The most represented sites were the femur (24, 25.8%) and tibia (52, 55.9%). Other involved bones included humerus, radius, clavicle, and calcaneus. Seventeen COM (18.3%) were in the diaphyseal region, whereas the majority (76, 81.7%) occurred in the meta-epiphyseal area.

A total of 26 patients (28.0%) had significant local or systemic comorbidities (C-M Class B hosts). According to anatomic type, 31 cases were type I, 13 type II, 21 type III and 28 type IV.

Vancomycin was added to PerOssal^®^ in most cases (80, 86.0%). In 24 of these, it was combined with Rifampicin. Rifampicin was added as a unique antibiotic in 10 (10.8%) cases, while it was mixed with gentamicin in 1 case. Gentamicin was used alone in two cases.

Plastic surgical skin closure was needed in 7 patients with a tibia COM (7.5%) (5 free flaps and 2 local flaps).

In 32 (34.4%) cases, intraoperative cultures were negative, but COM was confirmed by histology. Staphylococci were the commonest organisms, with *Staphylococcus aureus* (SA) isolated in 39 (63.9%) patients (18 methicillin resistant—MR and 21 methicillin sensible—MS). Gram-negative bacteria were isolated in 7 cases in monomicrobial infection and in 5 cases in polymicrobial infection, often with a Gram-positive organism (usually MRSA).

**Figure 1:**
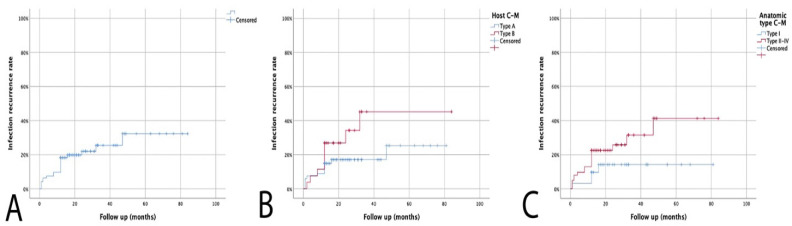
(**A**). Kaplan–Meier curve showing recurrence of infection rate. (**B**). Kaplan–Meier infection recurrence curve according to Cierny–Mader host subgroups. (**C**). Kaplan–Meier infection recurrence curve according to Cierny–Mader anatomic type.

Median follow-up was 21 months (range: 12–84). A total of 21 (22.6%) patients developed an IR after a median of 11 months (range: 1–47), giving an estimated IR rate of 18.3% at 1 year and 25.5% at 3 years of follow-up. (Figure 1A) All patients with recurrent infection had revision surgery with an eventual control of infection.

Further analysis of patients with a recurrence showed that failure to eradicate infection was significantly related to the physiological class of the host (*p* = 0.043) and anatomic type, with Type I CM having the lowest risk of IR (*p* = 0.047) (Figure 1B,C).

Nonetheless, IR was not significantly related to the isolated pathogen nor the local antibiotic therapy (*p* = 0.782 and *p* = 0.453, respectively). In particular, even MRSA infections had a similar outcome compared to other pathogens (*p* = 0.457).

Adverse events were uncommon. Eight patients (8.6%) had early leakage of fluid from wounds, which was actively treated and dried up without intervention or later recurrence of infection. None of the cases treated with a combined orthoplastic approach developed this complication.

Complete radiographic details were adequate to assess osseous repair in 58 cases (62.3%).

**Figure 2:**
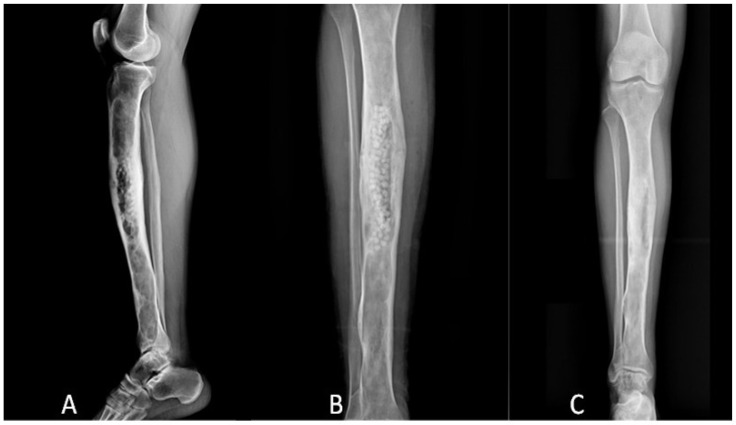
X-rays of the leg in a patient treated with debridement and adjuvant PerOssal bead for a chronic osteomyelitis of the tibia diaphysis. (**A**). Post-operative lateral view X-rays. Antero-posterior X-rays showing no filling at 6 months of follow-up (**B**) with complete filling at 12 months of follow up (**C**).

At 6 months, no filling of the defects was observed in 42 cases and a partial filling in 16 cases. However, at 12 months of follow-up, no filling of the defect was seen in 12 cases (20.7%), with partial filling in 24 cases (41.4%) and complete filling in only 22 cases (37.9%) (Figure 2).

## 4. Discussion

The surgical principles of COM may be interpreted as a combination of “radicalization” and “limitation”. The principle of “radicalization” requires the thorough removal of necrotic tissues and some adjacent healthy bone, to create a relatively clean wound, while the principle of “limitation” requires preserving as much healthy bone as possible to prevent the complication of postoperative fractures or deformities [24].

Debridement remains the mainstay of treatment, as highlighted by the extremely high recurrence of infection rates in the case of minimal debridement compared to wider debridement [25]. However, debridement alone without adequate dead space management is a sub-optimal approach in the management of chronic osteomyelitis. Bone void fillers should be considered a useful adjuvant therapy in COM, as highlighted by Chang et al. [26]. The authors compared the use of debridement alone with debridement and antibiotic-loaded CS pellets, observing a doubled recurrence with debridement alone compared to dead space management. Several studies have established the importance of obtaining a high local antibiotic concentration to reduce the rate of infection recurrence [12,16,26,27,28,29]. Many papers describing the use of various BVF (calcium sulphate, bioglass, PMMA, biocomposite granules, antibiotic-loaded demineralized bone matrix) have been published with extremely variable recurrence rates, ranging from 9% to 37.5% [12,16,29,30,31,32,33]. However, there is no clear evidence that any one approach to dead space management is superior.

To the best of our knowledge, only a few heterogeneous series have been reported for PerOssal^®^ [12,22,23]. Moreover, although reporting on relatively large cohorts, these studies altogether contained a variety of BVF.

Our results for the application of antibiotic loaded PerOssal^®^ are generally satisfactory. The eradication of infection rate is consistent with the results of many series (mostly small cohorts) of studies that discussed the usage of BVF [12,16,17,22,27,28,34]. Most likely, the reported failed cases were due to inadequate surgical debridement meaning that osteomyelitis was left unaddressed. PerOssal^®^ can only be integrated into vital bone, and thus a thorough debridement of the affected region should be performed prior to implantation. Nevertheless, even for patients with infection recurrence, a chance for re-debridement could be preserved and managed accordingly. We observed a lower infection recurrence rate in case of type I (medullary osteomyelitis), most likely because of a more feasible and aggressive curettage. Moreover, the better local control observed in type A hosts had already been described in a review by Pincher et al. [34]. More extensive procedures might have been reserved for fitter patients because of concerns about surviving or systemic complications in unfit patients.

PerOssal^®^ exhibits osteoconductive properties to promote bone ingrowth into the defect. It enables loading with varying antibiotics and releases these antibiotics in a sustained manner. Nanoparticulate HA in combination with CS revealed higher porosity and higher water uptake compared to pure CS. This leads to a higher antibiotic uptake and the faster release ofgentamicin and vancomycin within the first days followed by a long therapeutic duration (several weeks to months) [10,35,36]. PerOssal^®^ enables the specific antibiotic loading of CS according to antibiograms. Considering isolated pathogens, which include Gram-negative bacteria, the need for the local and systemic delivery of antibiotics must be emphasized for such bacteria. Although other bone substitutes have these characteristics, hardened PerOssal^®^ beads can be soaked with antibiotics immediately before use. This precludes a potential inactivation of antibiotics via hardening procedures.

After wide debridement, a second priority is helping osseous repair by providing the scaffold needed for bone ingrowth. The inclusion of HA, which is not passively dissolved, may provide a longer-lasting scaffold for bone formation. This potentially enhances the osteoconductivity of CS, providing a crystalline structure along which osteoblasts easily and eventually achieve self-repair without autogenous bone grafts [31,37]. Overall, we observed the complete disappearance of PerOssal^®^ beads and gradual new trabecular bone formation in treated areas in most analyzed cases. A progressive resorption of PerOssal^®^ beads over time has already been observed [27,38]. The partial to complete bone filling observed in our study confirms these data, thus encouraging aggressive debridement. Moreover, no fracture was observed in our cohort. However, there was a wide variety in resorption interval. This likely depends on the volume of the threatened area, age of the patient, site of the osteomyelitis, local condition of bone and soft tissues, weightbearing status and infection status [23]. By using CS alone, Ferguson et al. [16] reported partial bone ingrowth in 59% of 195 cases but a complete filling in only 4.4%. Badie et al. [39] described encouraging results using CS beads addicted with bone marrow aspirate, whereas in the series by McKee et al. [32] 9/25 patients needed a second surgery with bone graft positioning to achieve bone healing. In this aspect, calcium phosphates are superior to the CS as they take longer to dissolve [40,41,42]. Zhao et al. [43] directly compared CS and CS + BVF in calcium phosphates, reporting statistically higher new bone formation in the latter group. However, they conducted a completely different evaluation of bone filling, thus making any comparison to our data impossible.

In seven cases affecting the tibia, soft tissue coverage was needed [12,16,19,44,45,46,47]. Previous studies demonstrated that free flaps could increase blood flow and the delivery of antibiotics, enhance phagocytic activity and reduce bacterial counts [48]. Free flaps can also help to expedite bone healing in the early phases of repair [49]. The choice between muscle or fasciocutaneous flaps should be based on the size, depth, location of the wound, and length of the pedicle required [50].

Prolonged aseptic drainage was the only recorded complication in our study, with a relatively high rate of 8.6%. This incidence varied from person to person, primarily depending on the volume of implanted CS and soft tissues coverage. Previous studies on antibiotic-loaded CS reported prolonged wound drainage affecting 15% to 32% of patients [12,16,51]. Calcium sulphate dissolution leads to an acidic microenvironment responsible for local inflammatory processes at the site of implantation in human bone [32,52,53]. If not accompanied with typical presentations, positive inflammatory markers and imaging examination, prolonged aseptic drainage alone should not be considered as a sign of infection recurrence. It can be well-managed with regular dressing and wound care.

Some limitations must be acknowledged since this is a retrospective study. However, to the best of our knowledge, this is the largest series of patients affected by COM managed with a single-stage protocol, facilitated using PerOssal^®^ as a bioabsorbable BVF. Another limitation is the relatively short median follow-up, which does not allow for final conclusions, as COM can recur even many years after surgery. In addition, the heterogeneity of treated cases and the small size of each subgroup did not allow for any further analysis. Moreover, due to non-standardized radiographic follow-up, it was hard to analyze the bone filling of the defect. Finally, minimal data were reported regarding post-operatively prescribed systemic antibiotics, although regimens were tailored to an individual’s culture results. This large variation in practice makes it impossible to draw conclusions on the effectiveness of post-procedure antibiotic use.

Despite several bone void fillers can be used for local antibiotic delivery, PerOssal^®^ holds several practical advantages compared to other BVF. First, the addition of HA increases biocompatibility and reduces inflammatory reactions induced by biomaterials made from CS alone. Second, PerOssal^®^ can be loaded with a large range of antibiotics, based on previous culture samples and epidemiology data. Finally, it can be intra-operatively loaded with antibiotics. In conclusion, due to its good biocompatibility and antibiotic release, this composite material represents a viable adjuvant treatment in osteomyelitis.

## Figures and Tables

**Table 1:** Patient and treatment characteristics. MRSA: methicillin-resistant *S. aureus*; MSSA: methicillin-sensible *S. aureus*; CoNS: coagulase-negative staphylococci.

Characteristic	n (%)
Age, yeas (median, range)	40 years (4 to 73).
Sex	
Male	68 (73.1%)
Female	25 (26.9%)
Site	
Femur	24 (25.8%)
Tibia	52 (55.9%)
Humerus	6 (6.5%)
Radius	4 (4.3%)
Other	7 (7.l5%)
CM anatomic type	
I	31 (33.3%)
II	13 (14.0%)
III	21 (22.6%)
IV	28 (30.1%)
Local antibiotics Vancomycin Rifampicin Gentamycin Vancomycin + Rifampicin Rifampicin + Gentamycin	56 (60.2%) 10 (10.8%) 2 (2.1%) 24 (25.8%) 1 (1.1%)
Plastic surgery	
None	86 (92.5%)
Local flap	2 (2.1%)
Free flap	5 (5.4%)
Pathogen	
MRSA	18 (19.4%)
MSSA	21 (22.6%)
CoNS	10 (10.8%)
Enterobacteriaceae	7 (7.5%)
Polimicrobial	5 (5.3%)
Negative	32 (34.4%)

## Data Availability

The data presented in this study are available on request from the corresponding author.
